# Case Report: Diagnosis and management of primary malignant melanoma of the bladder: a case-based review

**DOI:** 10.3389/fonc.2026.1764989

**Published:** 2026-04-07

**Authors:** Xiaolong Hou, Xiaoxiao Xu, Yongxu Chen, Bohao Jiang, Yitong Xu, Hao Zhang

**Affiliations:** 1 Department of Urology, The First Hospital of China Medical University, Shenyang, China; 2 Department of Pathology, The First Hospital of China Medical University, Shenyang, China

**Keywords:** bladder melanoma, case report, melanoma, review, treatment

## Abstract

**Background:**

Primary malignant melanoma of the bladder (PMMB) represents an extraordinarily rare diagnostic entity among bladder malignancies, with fewer than 50 well-documented cases reported in modern medical literature. Due to its rarity, standardized treatment protocols remain undefined, and prognostic outcomes are poorly characterized.

**Case presentation:**

A 65-year-old man presented with a primary complaint of urinary tract irritation symptoms and an occupying lesion on the bladder wall detected during an enhanced CT scan. He underwent partial cystectomy and left ureterovesical reimplantation, and pathological results confirmed the diagnosis of PMMB. The prognosis was favorable, with no recurrence observed during a 24-month follow-up. Additionally, a comprehensive literature review was conducted to elucidate the typical characteristics and current therapeutic approaches reported to date, as evidence-based guidelines have yet to be established.

**Conclusion:**

Although PMMB is a rare bladder malignancy, certain similarities in its clinical and imaging features have been observed among the reported series. This study provides an additional summary of the tumor’s biological characteristics while discussing current treatment paradigms, future perspectives, and potential therapeutic strategies.

## Introduction

Melanoma, most of which originate from the skin, is recognized as the deadliest type of skin malignancy (1). It is highly aggressive and characterized by uncontrolled proliferation of melanocytes (2). Although distal metastasis is common, primary melanoma of the bladder (PMMB) remains extremely rare, accounting for less than 0.2% of all reported cases, with only around 40 reported cases so far (3, 4). Given its unique growth location, multiple surgical methods are currently employed for clinical decision-making, depending on tumor size and depth of invasion, including transurethral resection (TUR), partial cystectomy (PC), and radical cystectomy (RC), but with a poor average survival of less than 3 years.

Due to its rarity and atypical symptoms, standardized treatment approaches for both localized and advanced cases are currently lacking (5, 6). Herein, we report a PMMB case in detail to share our therapeutic experience and subsequently conducted a literature review to summarize published articles, aiming to deepen understanding of the disease and provide a basis for establishing a treatment consensus.

## Case presentation

**Figure 1:**
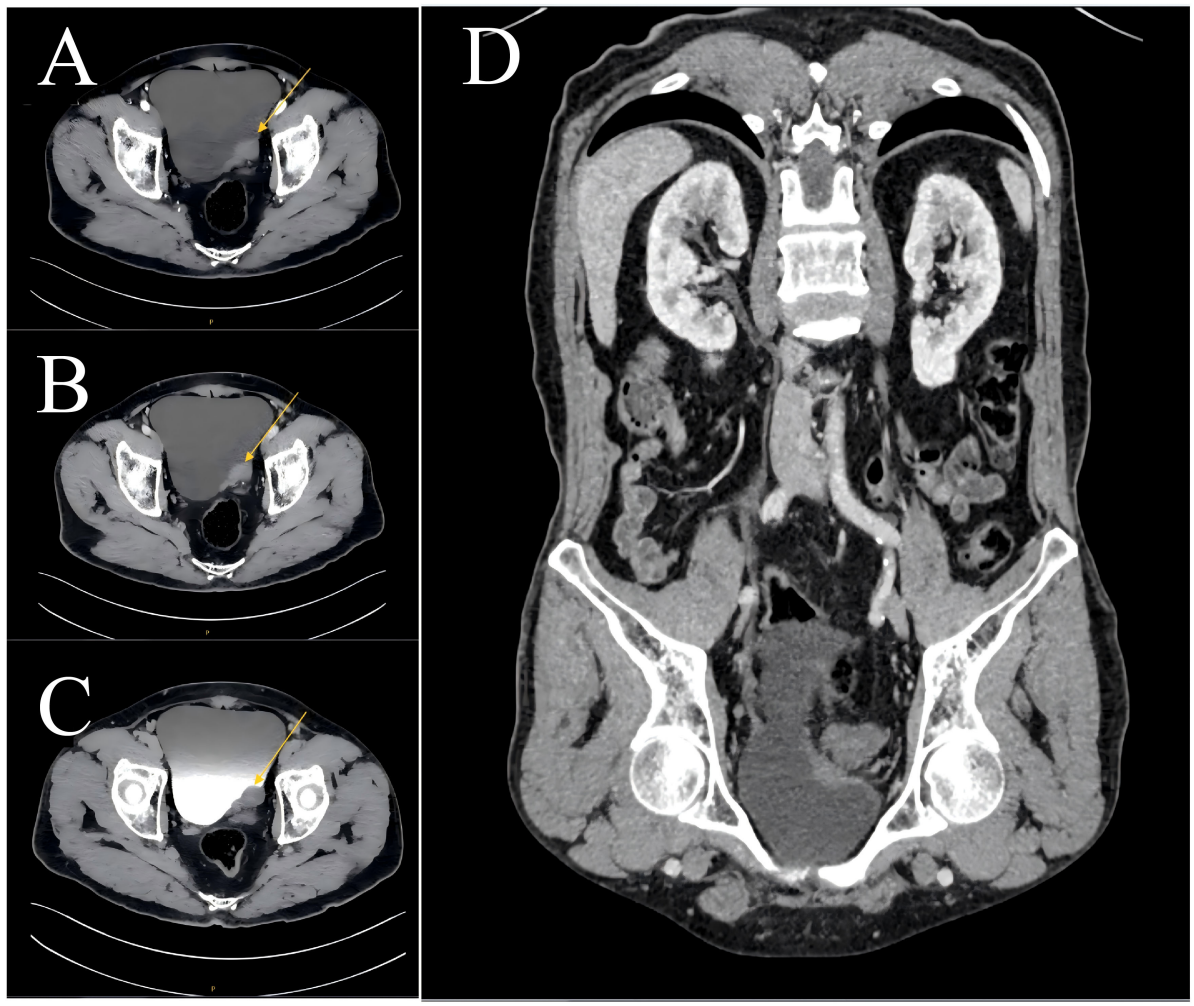
CT images of the male patient before surgical treatment. An irregular mass shadow (yellow arrow) measuring approximately 4 cm × 2 cm is visible at the left ureteric orifice, with adjacent bladder wall thickening and enhancement. **(A)** Arterial phase in the transverse position; **(B)** Venous phase in the transverse position; **(C)** Delayed phase in the transverse position; **(D)** Coronal CT manifestation.

A 65-year-old man presented to our outpatient clinic with lower urinary tract symptoms lasting 1 month, including urinary frequency, urgency, and dysuria. He denied any personal or family history of melanoma or other skin diseases, as well as any history of occupational or environmental exposure to chemical carcinogens. Urological ultrasound revealed a space-occupying lesion in the bladder, accompanied by left ureteral obstruction and ipsilateral hydronephrosis. Contrast-enhanced CT urography demonstrated an irregular mass (4.2 cm × 1.9 cm) near the left ureterovesical junction, with a baseline attenuation of 40 HU and mild heterogeneous enhancement (61 HU) during the contrast phase, along with adjacent bladder wall thickening and enhancement (**Figure 1**).

**Figure 2:**
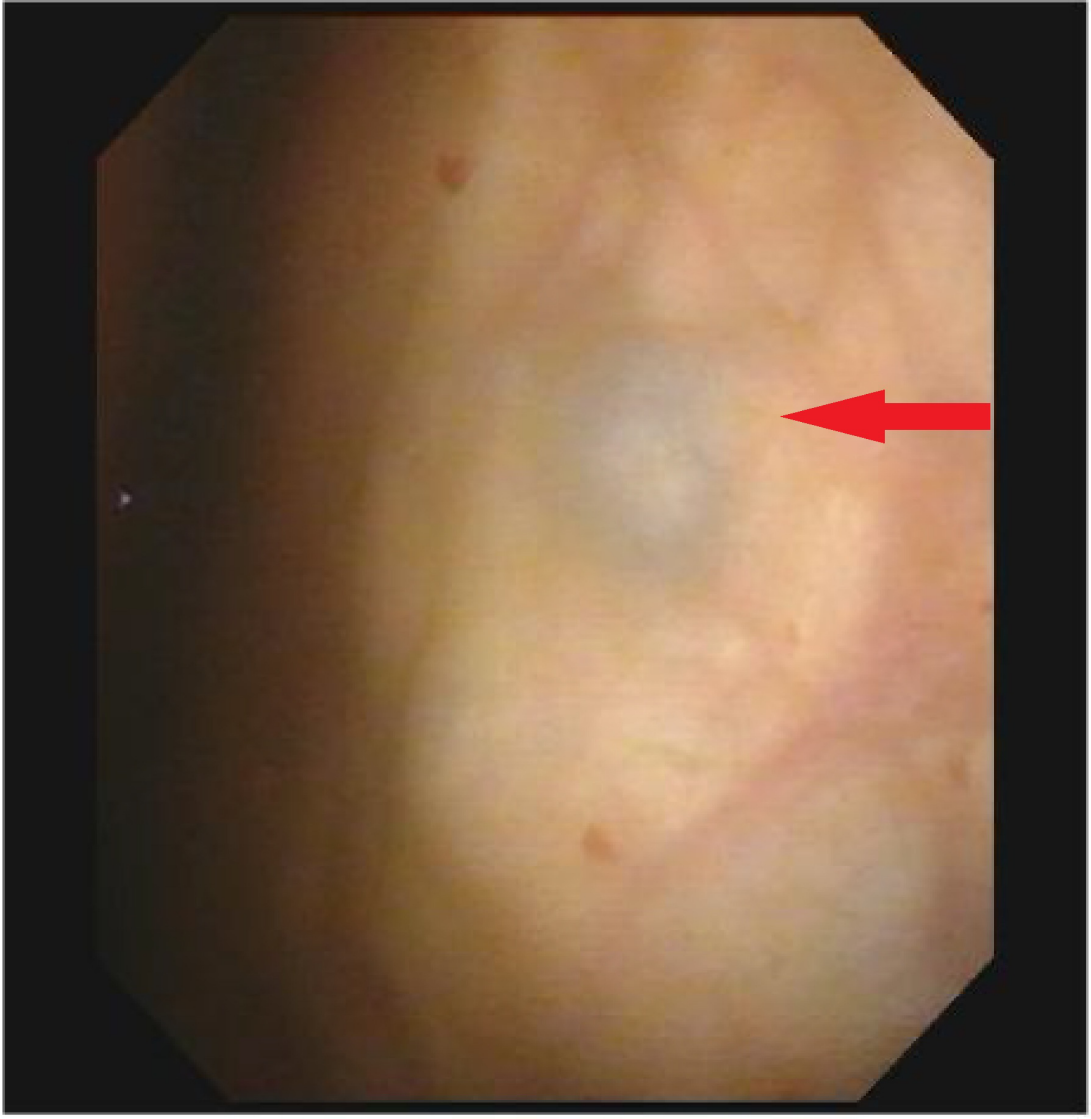
The appearance of the mass under cystoscopy. A blue follicular-like lesion measuring approximately 0.5 cm × 0.5 cm is visible in the submucosal layer of the left posterior bladder wall.

Cystoscopic evaluation identified a lobulated mass (4.0 cm × 3.0 cm) with relatively preserved surface mucosa, potentially originating from the muscularis propria, involving the left ureteral orifice, and demonstrating poorly defined margins with surrounding normal mucosa. A distinctive 0.5 cm × 0.5 cm submucosal blue follicular lesion was noted on the left posterior bladder wall, while the remaining bladder mucosa appeared normal. These findings were highly suggestive of malignant neoplasia (**Figure 2**).

Given the tumor’s involvement of the left ureteral orifice, with preservation of the muscularis propria on imaging, and the patient’s strong preference for bladder conservation, we elected to perform a partial cystectomy with left ureteroneocystostomy after comprehensive multidisciplinary evaluation to achieve negative margins while maintaining urinary continence through ureteral reimplantation.

After positioning the patient supine with the lower abdomen slightly elevated, a midline infraumbilical incision was made through the skin and subcutaneous tissues. The anterior rectus sheath was incised, and the rectus muscles were separated to expose the peritoneal cavity. The peritoneum was carefully reflected superiorly to achieve optimal bladder exposure. Two 7–0 silk traction sutures were placed on the bladder wall for retraction. Bladder entry was confirmed by aspirating clear yellow urine through needle puncture. A longitudinal cystotomy was performed, and the bladder was evacuated using suction. Intraoperative inspection revealed a tumor involving the left lateral bladder wall and bladder neck region, with no direct visualization of the left ureteral orifice. The left ureter was meticulously dissected extravesically. *En bloc* resection was performed with 2 cm macroscopic margins, including the involved bladder wall and intramural ureteral segment. The bladder defect was closed in a watertight fashion using running 3–0 absorbable sutures. A new ureteral orifice was created at the bladder dome through a stab incision, where the mobilized ureter was spatulated (0.5 cm) and anastomosed to the bladder mucosa with eight interrupted 3–0 absorbable sutures in a full-thickness manner. To prevent postoperative stricture formation, a 4.7-Fr double-J stent was placed in the left ureter. Additional reinforcement was achieved by approximating the ureteral and bladder muscularis with 4–0 absorbable sutures at the anastomotic site. After confirming complete hemostasis, a closed-suction drain was placed in the perivesical space, and the abdominal wall was closed in anatomical layers.

**Figure 3:**
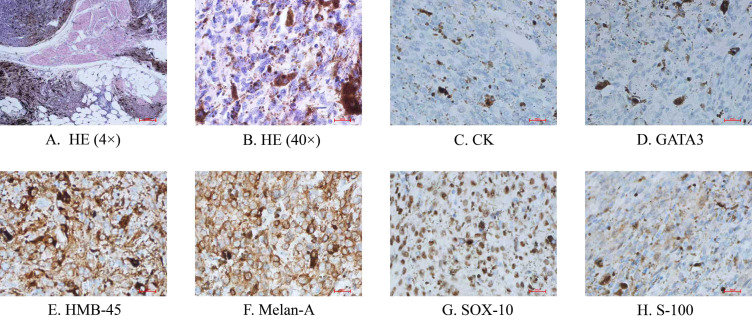
Pathological manifestations of the tumor with IHC staining. **(A)** HE staining (× 4); **(B)** HE staining (× 40); **(C)** CK staining; **(D)** GATA3 staining; **(E)** HMB-45 staining; **(F)** Melan-A staining; **(G)** SOX-10 staining; **(H)** S-100 staining.

Histopathology confirmed malignant melanoma. HE staining (**Figures 3A, B**) reveals tumor infiltration into the muscular layer of the bladder, adventitia, and surrounding adipose tissue. The cells exhibit an epithelioid morphology with marked atypia, characterized by large nuclei, prominent nucleoli, distinct chromatin, and increased mitotic figures. Abundant melanin granules are observed both intracellularly and within the stroma. Histopathology confirmed malignant melanoma, supported by immunohistochemistry. Immunohistochemical staining shows CK(−) (**Figure 3C**), PAX8(−), GATA3(−) (**Figure 3D**), CK7(−), and CK20(−), ruling out anepithelial-derived tumor. Combined with HMB-45(+) (**Figure 3E**), Melan-A(+) (**Figure 3F**), MITF(+), SOX-10(+) (**Figure 3G**), and S-100(+) (**Figure 3H**), these findings confirm the diagnosis of melanoma. The PD-L1 (Ventana SP263) test result shows a Tumor Proportion Score (TPS) of 60% (**Figure 3**).

**Figure 4:**
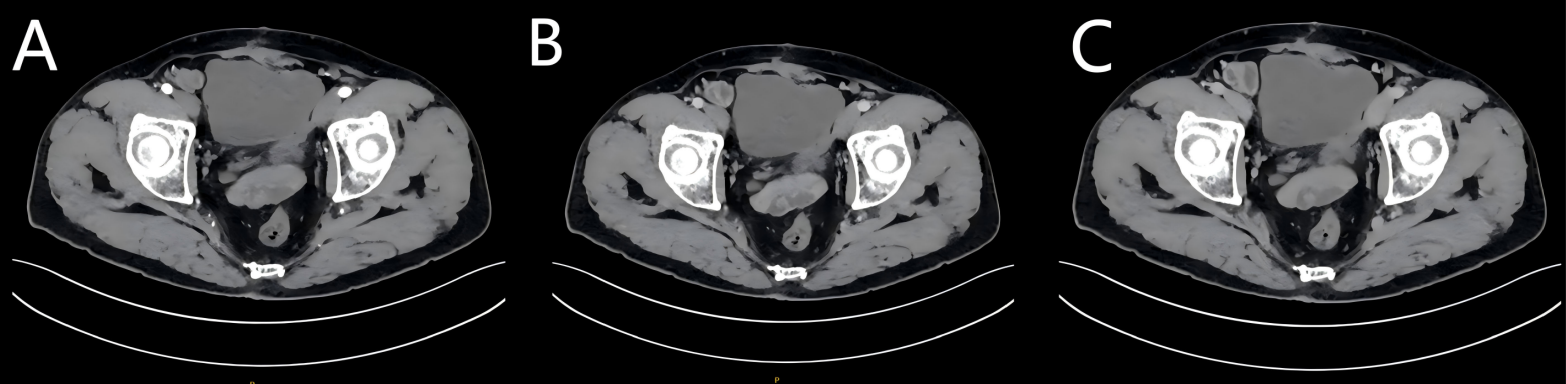
Enhanced CT examination at the 6-month postoperative follow-up. The images show normal postoperative manifestations with a uniform enhancement. **(A)** Arterial phase; **(B)** Venous phase. **(C)** Delayed phase.

The patient recovered uneventfully postoperatively and was discharged on the eighth day after surgery. The remaining double-J stent was removed 2 months later. Owing to the rarity of the diagnosis, Kirsten rat sarcoma viral oncogene (KRAS)/Neuroblastoma ras viral oncogene (NRAS)/Phosphatidylinositol-4,5-bisphosphate 3-kinase catalytic subunit alpha (PIK3CA)/B-Raf proto-oncogene (BRAF) genotype sequencing with adjuvant cisplatin-temozolomide chemotherapy was recommended, but it was declined due to financial constraints. The required follow-up examination was performed 6 months after surgery, showing normal postoperative findings on enhanced CT, which revealed a locally incomplete bladder contour and uniform enhancement (**Figure 4**).

**Figure 5:**
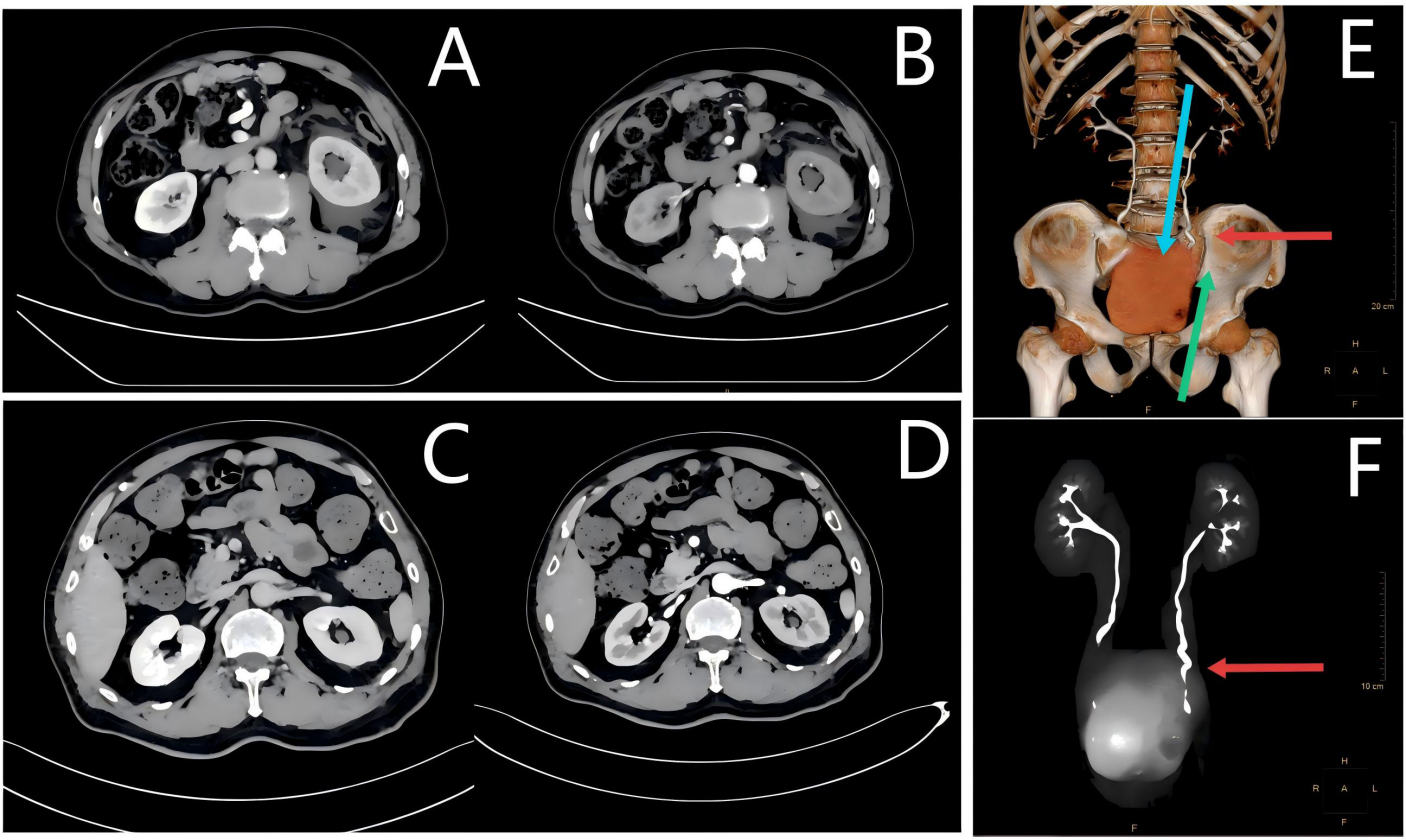
CT images before and after the covered stent implantation. **(A, B)** Enhanced CT reveals left-sided hydronephrosis with contrast retention on the left side. **(C, D)** Enhanced CT scan indicates significant resolution of hydronephrosis in the patient’s left kidney. **(E)** Urinary tract 3D reconstruction and a schematic diagram of the route for the dual-scope surgical protocol. The red arrow shows the “Z”-shaped structure. During dual-scope coordination, the blue arrow indicates the nephroscope, while the green arrow indicates the ureteroscope. **(F)** Computed tomography urography. The red arrow shows the “Z”-shaped structure.

However, the patient reported left flank pain 8 months after surgery, with CT findings showing dilatation and hydronephrosis of the left renal pelvis and ureter (**Figure 5**). During the subsequent ureteroscopy, repeated attempts to locate the anastomotic site of the ureteral reimplantation were unsuccessful, even with a flexible cystoscope. Left percutaneous nephrostomy was performed to alleviate hydronephrosis. The patient was readmitted and underwent cystoscopy after relief of hydronephrosis. Positioned in the right lateral decubitus lithotomy position, the left ureteric orifice remained undetectable. With another flexible ureteroscope introduced through the left renal approach, dual-scope coordination ultimately localized the needle-point-sized site of the reimplanted left ureteric orifice. Under guidewire guidance, the stenotic segment, forming an extremely challenging “Z”-shaped stricture, was dilated, and a covered stent was successfully implanted. An F4.7 double-J stent, along with a left nephrostomy tube, was left in place and removed 1 month later. As of 30 September 2025, the patient was confirmed alive without evidence of recurrence or metastasis through a telephone follow-up 24 months after partial cystectomy. A timeline figure was created to present the case history more clearly and intuitively (**Supplementary Figure 1**).

## Patient perspective

Initially, severe urinary symptoms significantly disrupted my life. I was shocked by the rare diagnosis of primary bladder melanoma, especially given my complete lack of personal or family history of melanoma. Because of my strong will of preserving bladder, I was greatly reassured by the team’s decision to perform a bladder-sparing partial cystectomy. My early recovery was smooth. Although financial constraints prevented me from pursuing adjuvant therapy and genetic testing, I was highly satisfied with the initial outcome.

After 8 months, sudden severe left flank pain and hydronephrosis caused renewed anxiety. Following a temporary percutaneous nephrostomy, the medical team successfully bypassed a complex “Z”-shaped stricture using a dual-scope approach and implanted a stent. This intervention rapidly relieved both my physical pain and psychological burden.

At 24 months post surgery, I am deeply grateful to remain cancer-free, with no signs of metastasis. Overall, I am highly satisfied with the successful preservation of my bladder and my excellent overall recovery.

## Discussion

Melanoma, typically arising in cutaneous tissue, is known for its high invasiveness, lethality, and metastatic potential (7). Its pathological basis lies in the malignant transformation of melanocytes, cells derived from the embryonic neuroectoderm and widely distributed in tissues such as the skin, ocular choroid, mucosa, and rectum. Monocytes perform the biological function of protecting against light-induced damage by producing melanin (8). Their widespread distribution provides the histological basis for the occurrence of primary melanoma in various tissues throughout the body. However, the bladder is an extremely rare primary site, accounting for less than 1% of melanomas (3). Considering the rarity of its growth location, the understanding of the diagnosis and treatment of PMMB remains limited, and no guidelines are available to provide precise management recommendations.

Multiple articles have previously summarized reported case reports, but these are limited by their publication dates and have not provided a detailed comparison of different treatments and postoperative outcomes. To address this gap, we conducted a comprehensive literature review using the following search query: (“Primary Melanoma of the Bladder”[Title/Abstract] OR “Primary Bladder Melanoma”[Title/Abstract] OR “Melanoma of the Bladder”[Title/Abstract] OR “Bladder Melanoma”[Title/Abstract]) AND (“Case Report”[Publication Type] OR “Case Series”[Publication Type] OR “case report”[Title/Abstract] OR “case series”[Title/Abstract]). Forty-three cases of PMMB were retrieved from PubMed, EMBASE, and Web of Science databases since Wheelock and colleagues first reported the initial case in 1942. All case outcomes are summarized in **Table 1**.

**Table 1:** Summary of all reported cases from our literature review.

Year	Reference	Gender	Age	Chief complaint	Location	Size (cm)	Treatment	Postoperative stay	Recurrence	Follow-up (months)	Outcomes	Stage	Adjuvant therapy	IHC	Postoperative complications
1942	Wheelock (9)	Woman	67	Gross hematuria	Bladder wall and urethra	5	PC	23 days	–	36	Died	–	–	–	–
1962	Prince (10)	Woman	61	Painless gross hematuria	Dome	0.5	Single deep bite with resectoscope	< 8 weeks	Generalized metastases	2	Died	–	–	–	–
1976	Ainsworth (11)	Woman	65	Painless gross hematuria	Anterior wall	6–8	RC	–	Incisional and pelvic tumor (after 14 months)	17	Alive	–	BCNU and Vincristine pelvic irradiation (2,750 R in two separate courses)	–	–
1980	Willis (12)	Woman	57	Bladder irritation	Bladder neck	–	RC	37 days	Metastases (after 3 years)	36	Died	–	Intravesical BCG and RT	–	Renal failure and bowel obstruction
1982	Anichkov (13)	Man	48	Periodic macrohematuria	Fundus	5 × 4	PC	2 weeks	Metastases in both lungs (after 1 year)	12	Died	–	–	–	–
		Man	46	Gross hematuria	Ostium of the ureter	3 × 3	RC	–	–	3	Alive	–	–	–	–
1985	Ironside (14)	Man	56	–	–	–	None	–	Metastases	8	Died	–	RT	–	–
1988	Goldschmidt (15)	Woman	53	Gross hematuria	Lateral wall	–	PC	–	–	6	Alive	–	–	–	–
		Woman	56	–	–	–	None	–	Metastases in the lung and liver	7	Died	–	–	–	–
1989	Philippe (16)	Man	77				TUR			–	–				
1992	Ahlen (17)	Man	81	Bladder irritation	Trigone	–	RC	–	Recurrence after 3, 6, and 8 months	24	Alive	–	Interferon-α and RT	–	–
1992	Lund (18)	Woman	81	Hematuria	Anterior wall	–	Local excision	–	–	15	Alive	–	Chemotherapy and RT	–	–
1992	Kojima (19)	Woman	63	Melanoma metastases	–	–	TUR	–	Metastases	18	Died	–	–	S-100, NSE, Leu-7, HMB-45	–
1993	Lange-Welker (20)	Man	75	Disturbed micturition and gross hematuria	Diverticulum on the left bladder wall	fist-sized	PC	–	Intracranial metastasis after 6 weeks	3	Died	–	RT	S-100	–
1993	Mourad (21)	Man	34	–	–	–	RC	–	–	12	Alive	–	–	–	–
1993	Lome (22)	Man	53	Bladder irritation	Fundus	–	RC	–	–	18	Alive	–	–	–	–
1995	Torres (23)	Man	44	Gross hematuria	Fundus and anterior right wall	8 × 6.5	RC	–	–	14	Died	–	–	–	Enterocutaneous and enterourinary tract fistulas
1999	Tainio (24)	Man	52	Gross hematuria	Fundus of the bladder	–	TUR	–	–	8	Died	–	Interferon and cytostatic	S-100 and HMB-45	–
2000	Montes (25)	Woman	44	Gross hematuria	Right wall	2 × 3	TUR	–	–	144	Alive	–	–	S-100	–
2001	Khalbuss (26)	Woman	82	Gross hematuria	–	–	RT	–	13 weeks after the operation	16	Died	–	RT: external irradiation (3,600 rad) to the pelvis	–	Wound infection and thrombosis
2002	Hsu (27)	Man	73	–	–	–	TUR	–	–	16	Alive	–	Intravesical BCG	–	–
2005	Baudet (28)	Woman	7	–	–	–	PC	–	–	84	Alive	–		–	–
2006	Pacella (29)	Man	82	Gross hematuria	Posterior wall	–	TUR	–	Metastases after 12weeks	9	Died	–	–	S-100 and HMB-45	–
2011	Sundersingh (30)	Man	56	Gross hematuria	Anterior wall	9 × 6 × 6	RC	–	Pelvic recurrence after 16 weeks	10	Died	–	–	S-100, HMB-45, and Melan-A	–
2011	Ammari (31)	Man	71	Gross hematuria	Trigone	5 × 4 × 1	TUR	–	–	5	Died	–	–	–	–
2013	Truong (32)	Woman	84	Gross hematuria	–	–	TUR	–	Metastases after 20 weeks	–	–	T3N0M1	Ipilimumab	S-100 and Melan-A	–
2016	Karabulut (33)	Man	52	–	Left wall	3.5 × 3 × 1.5	RC	–	–	60	Alive	–	–	S-100, HMB-45, and Melan-A	–
		Woman	63	Hematuria	Apex	2 × 2 × 1.6	RC	–	–	12	Alive	–	–	S-100, HMB-45, and Melan-A	–
		Woman	76	Hematuria	–	–	TUR	–	–	15	Alive	–	Vemurafenib 2 × 960 mg/day	S-100, HMB-45, and Melan-A	–
		Man	54	Dysuria	–	3 × 2.5 × 2.5	TUR	–	–	4	Died	–	–	S-100, HMB-45, and Melan-A	–
		Man	70	Hematuria	–	–	None	–	–	32	Died	–	–	S-100, HMB-45, and Melan-A	–
2017	Otto (34)	Man	52	–	–	–	TUR	–	–	18	Died	–	Interferon/Dacarbazine	S-100, HMB-45, and Melan-A	–
2018	Barillaro (35)	Man	72	–	–	–	RC	–	–	16	Alive	–	Nivolumab	–	–
2019	Bumbu (36)	Man	80	Macroscopic hematuria	Prostatic urethra and left wall	–	TUR	–	–	6	Died	T2N1M0	–	–	–
2019	Kirigin (37)	Woman	87	Gross hematuria and urinary incontinence	–	–	None	–	–	0.5	Died	–	–	CK7, CK20, PAN-CK, S-100, HMB-45, tyrosinase, and MITF	–
2019	Mercimek (38)	Woman	39	–	–	–	PC	–	–	52	Alive	–	–	–	–
2019	Chaus (39)	Woman	27	Gross hematuria	Right anterior wall	1.9 × 1.7 × 2.6	Robotic PC	–	–	24	alive	T1N0M0	Pembrolizumab	SOX, S-100, Melan-A/MART1, HMB-45	–
2020	Rubio (40)	Woman	39	Gross hematuria	–	–	TUR		Metastases	1.5	Died		Temozolomide	S-100, HMB-45, and Melan-A	–
2021	Snajdar (41)	Woman	78	Gross hematuria	–	–	RC	–	Metastases	14	Died	T3N0M0	–	S-100 and HMB-45	–
2021	Rapisarda (42)	Woman	74	Gross hematuria	–	–	TUR	–	–	6	Alive	T1N0M0	Intravesical BCG	S100 and MART-1/MELAN-A	–
2021	Quaquarini (43)	Woman	81	–	Trigone	–	PC	–	–	14	Alive	T4N1M0	–	Mart-1, HMB-45, and S-100	–
2022	Dai (44)	Woman	67	Gross hematuria	Bladder neck	1	RC	–	–	15	Alive	–	–	Mart-1, HMB-45, and S-100	–
2025	Kayraklioglu (45)	Woman	70	Gross hematuria	Bladder neck and orifice	1.9 × 1.5	RC		Metastases	43	Died	–	–	SOX10, Melan-A, HMB-45	–

TUR, transurethral resection; BCG, Bacillus Calmette–Guérin; PC, partial cystectomy; RC, radical cystectomy; RT, radiation therapy.

In terms of demographic characteristics and typical symptoms, middle-aged and elderly people are more susceptible, with no significant difference in incidence between men and women. According to our review, the median age of patients was 63 (from 7 to 87) years old, and 20 patients were men (20/43). The age distribution was concentrated among patients aged 50 and above, with 34 cases. Most patients were admitted primarily for gross hematuria, while one patient presented with metastatic melanoma at another body site as the initial diagnosis (19). Patients usually seek medical help due to symptoms of gross hematuria and bladder irritation, which are atypical among bladder malignancies (12, 22). Some patients may experience pain due to tumor invasion of the urethra or surrounding tissues (36).

PMMB is also similar to urothelial carcinoma in imaging, often manifesting as partial thickening and uneven enhancement of the bladder wall, with invasion into surrounding tissues in some cases. Sometimes, melanin deposition within the mass may present an abnormal local signal (46). Cystoscopy is an intuitive and noninvasive method used for preoperative diagnosis and has important value in cases of PMMB. The tumors primarily grew within the bladder wall, with 1% of patients experiencing tumor invasion into the ureter and urethra. Originating from melanocytes, the pigmented masses often present a brownish-gray or black appearance, and bleeding may sometimes be observed. However, the color can be misleading during diagnosis between PMMB and urothelial carcinoma, as PMMB may occasionally exhibit melanin deficiency (43).

Currently, pathology examination remains the gold standard for accurate diagnosis due to atypical symptoms and growth patterns. In terms of gross morphology, PMMB usually presents as brownish-gray or black nodules, which are prone to invade the lamina propria or muscular layer (47). The cell morphology exhibits diversity, with epithelioid or transparent cells under the microscope and visible melanin granules in the field of view. PMMB usually demonstrates a characteristic immunohistochemical profile, with positive melanin markers such as SOX10, S-100, Melan-A, HMB-45, and PRAME (48). GATA3 and CK7/20, which may aid in distinguishing it from urothelial carcinoma, are usually negative (7).

Urothelial carcinoma aside, PMMB should also be differentiated from melanoma, which metastasizes to the bladder wall (49). The rules for distinction include a history of prior skin lesions, evidence of cutaneous or visceral malignant melanoma, and atypical melanocytes at the tumor margin (50). Another distinguishing point is bladder melanosis, which results from the accumulation of macrophages containing melanin in the bladder mucosa. It is generally considered a benign condition, with no abnormal tumor cells observed under the microscope, and it typically presents no obvious clinical symptoms. It is often accidentally discovered during cystoscopy and may be confused with PMMB because of its pigmented appearance (51).

Due to the malignancy and metastatic potential of PMMB, timely and appropriate treatment is crucial. Thorough resection of the tumor with a clean margin remains the key to effective treatment. Our review indicates that most patients (38/43) underwent surgical treatment, with approaches including transurethral resection, partial cystectomy, and radical cystectomy; the growth pattern and depth of invasion determine the choice of method. Careful preoperative evaluation should assess lymph node involvement and distal metastasis to guide adjustments to the surgical plan, such as regional lymph node dissection (52). Only four articles published in the last century explicitly reported postoperative discharge time, with an average length of stay of 32.5 days. However, a significant difference was observed in our reported case, possibly due to improvements in medical and nursing techniques over the past half-century. Among the 43 cases, eight patients underwent partial cystectomy, 14 underwent radical cystectomy, and 16 underwent TUR. Five patients did not undergo surgical treatment for various reasons. Notably, our survival analysis of overall survival for these surgical protocols indicated no significant differences in prognosis (**Supplementary Figure 2**). This emphasizes that, under the premise of complete tumor resection, less invasive methods may be equally effective in ensuring prognosis. With the development of surgical robots, robot-assisted surgery has also been applied to the treatment of PMMB. Chaus’s team reported a case of robot-assisted partial cystectomy, with an uneventful prognosis during follow-up (39). However, there are still too few reported cases of similar treatment, and it remains unknown whether robot-assisted surgery offers potential benefits.

In addition to surgical treatment, some cases also received adjuvant therapies. The patient in Hsu’s report received intravesical *Bacillus* Calmette–Guérin (BCG) after surgery, which is a common method for reducing recurrence in urothelial carcinoma. However, there is currently insufficient evidence to support the efficacy of this treatment for PMMB. The treatment experience of melanoma in other locations also informs adjuvant treatments. In some cases, PMMB patients received FDA-approved systemic therapy for metastatic melanoma, including chemotherapy (dacarbazine), cytokine (interferon alfa), and PD-1 inhibitors (nivolumab and ipilimumab). According to our review, 13 patients received postoperative chemotherapy with the previously mentioned regimen out of the 43 cases. Among them, eight patients remained alive during the follow-up period. Considering all treatment cases and multidisciplinary consultation, as well as drawing on the treatment experience of cutaneous melanoma, appears to be a promising approach for PMMB with high risk or confirmed distant metastasis.

To date, few published reports exist on genetic testing results for bladder melanoma. The rarity of PMMB cases poses a significant challenge for directly exploring the genetic mutation characteristics and corresponding targeted therapies. The treatment experience of melanoma at other sites may serve as a reference, such as cutaneous melanoma and mucosal melanoma at other sites may serve as a reference, such as cutaneous melanoma and mucosal melanoma, where gene profiling has demonstrated growing importance (53). In addition to common characteristic mutations (BRAF/NRAS/KIT proto-oncogene (KIT)), next-generation sequencing and whole-genome sequencing have revealed other highly mutated genes, including KRAS, PIK3CA, TP53, SF3B1, and OXA1L (54, 55). Retrospective trials have also shown differences in prognosis and therapeutic efficacy between different genotypes, and a comprehensive gene-sequence report may provide evidence for selecting a targeted therapy plan, such as BRAF, MEK, and KIT inhibitors (56–58).

Our present report has several strengths. First, compared with other articles that focus on symptoms and pathological features, this report provides a comprehensive report of the entire diagnosis and treatment process, including preoperative evaluation, CT and cystoscopy findings, surgical planning, intraoperative observation, and postoperative regular follow-up. Second, in addition to a sufficient follow-up period, we provide detailed reports on postoperative ureteral-bladder anastomotic stenosis and its treatments, accompanied by imaging data before and after surgical relief, thereby sharing our experience with potential postoperative adverse events and their management. Third, we have updated the literature review of PMMB to date and provided a more comprehensive summary of common growth sites and postoperative recovery situations, which are often overlooked but represent important details for clinical decision-making. We conducted a survival analysis on postoperative outcomes of different surgical methods. Our report, however, has limitations. In the survival analysis, statistical bias may have been introduced due to the varying severity among patients. Owing to the patient’s limited financial resources, molecular profiling and systemic therapy were not pursued postoperatively. Our review also revealed a notable shortcoming in the reporting of molecular profiling of PMMB in the existing literature.

## Conclusion

In summary, PMMB is an extremely rare malignancy that poses a significant challenge for diagnosis and treatment. Although it has unique yellow-black characteristics and exhibits higher invasiveness, it shares similarities with urothelial carcinoma and is difficult to distinguish based on symptoms, imaging, and cystoscopy. Accurate diagnosis still relies on pathology, and there is currently no consensus on treatment despite the poor prognosis. Current management strategies are largely based on experience with urothelial carcinoma and cutaneous melanoma, including complete resection, lymph node dissection guided by invasiveness, and adjuvant systemic treatments. Regular follow-up also plays a crucial role throughout the treatment course. Although we have summarized previously reported cases and shared our treatment experience, further studies are needed to establish a foundation for consensus on diagnosis and treatment in the future.

## Data Availability

The original contributions presented in the study are included in the article/**Supplementary Material**. Further inquiries can be directed to the corresponding authors.
