# Supplementary material for: Case Report: Diagnosis and management of primary malignant melanoma of the bladder: a case-based review
Source: Front Oncol. 2026 Apr 7;16:1764989. doi: 10.3389/fonc.2026.1764989 (PMC13095624; doi:10.3389/fonc.2026.1764989)

**Supplementary Figure 1. The Timeline of our Case from diagnosis to prognosis.**

**
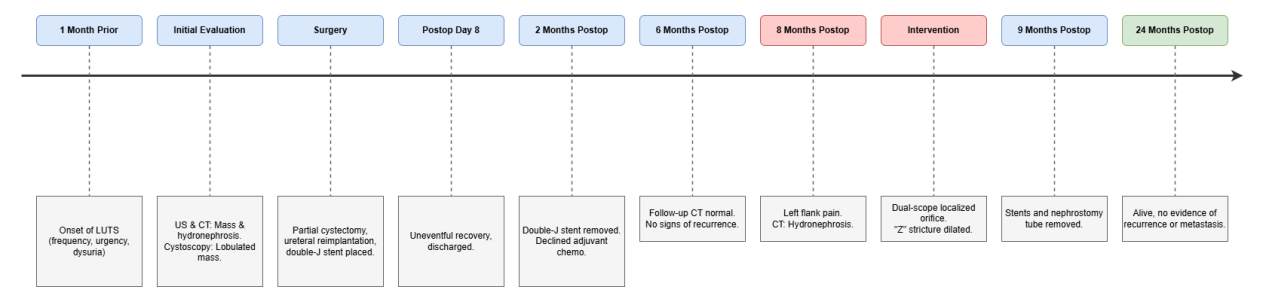
**

**Supplementary Figure 2.** The Kaplan-Meier survival curves for overall survival (OS) of all three surgical methods. (1) No significant differences were found in both overall and pairwise comparisons: P_overall_ = 0.065; P_PC-TUR_ = 0.354; P_RC-TUR_ = 0.145; P_PC-RC_ = 1. (2) Blue line (TUR): transurethral resection; Red line (PC): Partial cystectomy; Green line (RC): Radical cystectomy.


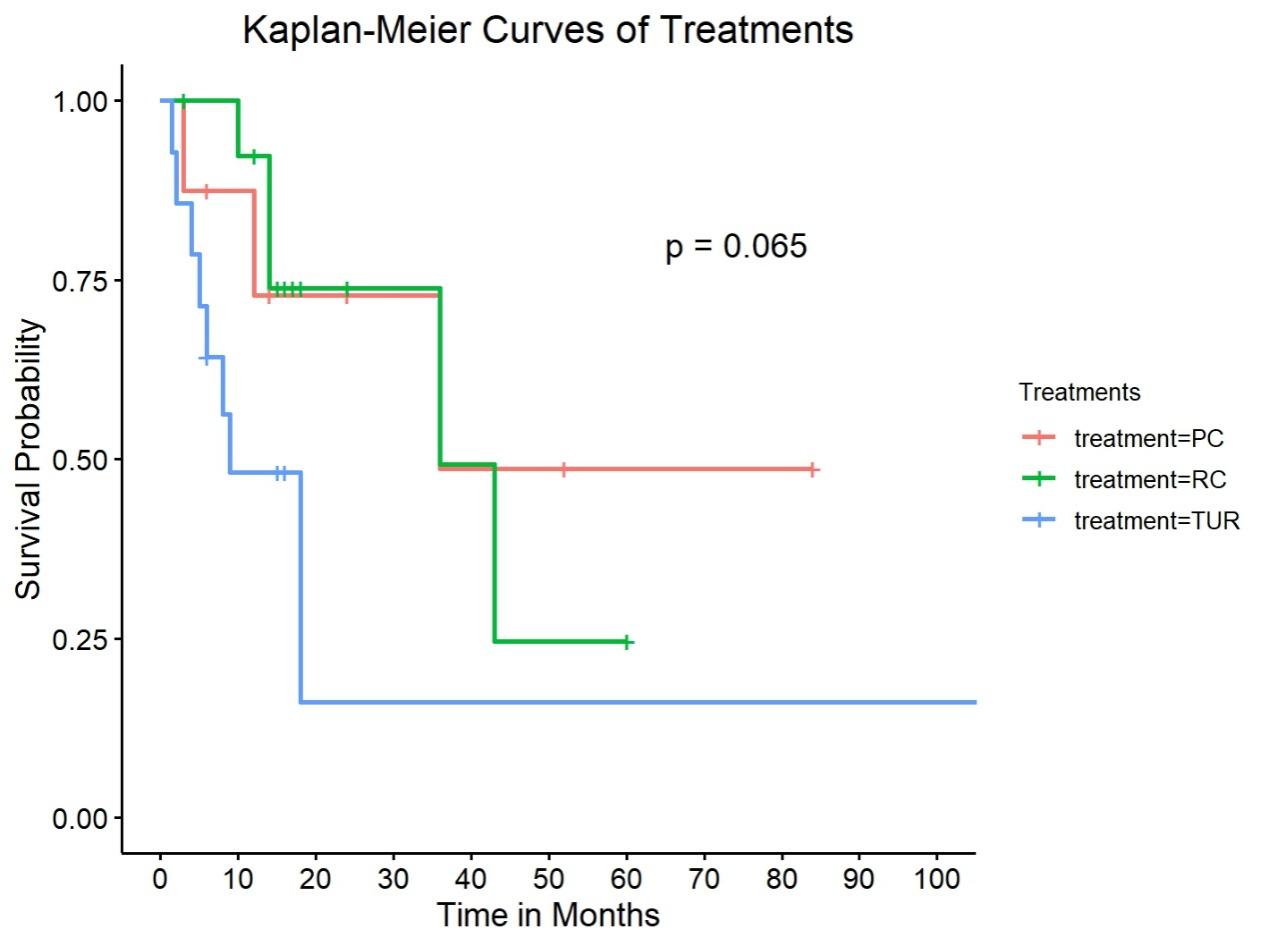

Supplement: Supplementary file 1 [file DataSheet1.docx]
